# Performance Nutrition for Athletes

**DOI:** 10.1007/s40279-018-1027-9

**Published:** 2019-01-22

**Authors:** Lawrence L. Spriet

**Affiliations:** Human Health and Nutritional Sciences, University of Guelph, Guelph, ON N1G 2W1 Canada

A sound nutritional plan is essential for achieving and maintaining optimal athletic performance. In addition to daily meal planning, a sports nutritionist pays special attention to the needs of athletes before, during and following training sessions and competitions. A wide variety of foods and nutritional products are available for the athlete to meet these needs. Sports nutrition professionals spend a lot of time reading and interpreting the relevant literature, and in many cases, researching the best products and ways to deliver the needed nutrients. In the research world, we often examine individual ingredients that have been removed from foods in an effort to examine the potential beneficial effects in isolation. In the real world, we most often eat foods that contain several important ingredients. So, it is ultimately also necessary to conduct research studies with real foods to determine how the food is received as a whole, and whether the important ingredients reach the target tissues in sufficient amounts, whether they interact with each other, and of course, whether beneficial effects are realized. This supplement examines the potential complications and benefits of eating foods in the context of achieving and maintaining optimal performance.

The Gatorade Sports Science Institute (GSSI) has been bringing sports nutrition and sports science researchers together for the past 30 years to address many issues that relate to the health, wellbeing and performance of athletes. Since 2012 this gathering has been known as the GSSI Expert Panel, which continued in 2017 with a meeting in October to discuss several nutritional issues that influence athlete performance. Following the meeting, the authors summarized the recent work in their topic area, resulting in the manuscripts in this Sports Medicine supplement (the sixth in a series supported by GSSI).

The first paper [1] addresses the intriguing topic of translating sports performance nutrition research into the real world and ultimately the chances of it helping an athlete maximize their performance to reach the “podium.” The authors present a framework they call the “Paper-2-Podium Matrix” which provides several criteria to critically evaluate performance nutrition-related research papers. In this manner, the sports nutrition practitioner can decide whether the research in question can be translated into something useful for the athletes they advise.

The second paper [2] in the supplement examines the very timely topic of foods that contain gluten or fermentable oligo-, di-, monosaccharides and polyols (FODMAPS) and their roles in producing gastrointestinal (GI) symptoms in athletes. The author suggests that the popularity of gluten-free diets (GFD) among athletes may not be warranted as research investigating the effects of a GFD in nonceliac athletes has not shown positive effects when compared to a gluten-containing diet on GI health, systemic inflammation, perceptual wellbeing or performance. It appears that a reduction in FODMAPs concurrent with the elimination of gluten-containing grains may be the modulating factor of GI symptom improvement.

The third paper [3] examines the possibility that phytochemicals may improve some aspects of a person’s cognitive function and psychological state and improve athletic performance. It appears from work in non-athletes that secondary metabolite phytochemicals from the main structural groups—phenolics (polyphenols), terpenes and alkaloids—may improve cognitive function and psychological state, which may be relevant for sports performance. However, this suggestion awaits more research in a sporting context before any solid practical recommendations can be made.

The next paper [4] of the supplement digs deeper into the roles that fruit-derived polyphenols may play in enhancing athletic performance and recovering from exercise-induced muscle damage. Polyphenols have antioxidant and anti-inflammatory properties and may enhance exercise performance by regulating the excess reactive oxygen species generation that have been implicated in fatigue development. Recovery from intensive exercise may also be improved by polyphenols by limiting inflammation and oxidative damage in muscle. While some research does exist to support these claims, more research is needed both from an efficacy and mechanistic perspective.

The fifth paper [5] discusses the importance of having “meal recommendations with protein-rich whole foods” to maximize post-exercise muscle protein synthesis rates rather than recommendations for the ingestion of isolated protein sources. Knowledge related to protein quality and the interactive effects with the food matrix is needed to achieve optimal protein requirements during the post-exercise recovery window.

The final paper [6] examines how exercise and hot environments can lead to varying levels of mild dehydration if the important and most basic of nutrients, water, together with electrolytes, are not consumed in adequate quantities. The authors describe how dehydration can impact the physiological functioning of the human heart, muscles and brain differently during exercise requiring both low and high functional demands.

It is clear in the papers of this supplement that sports nutrition research has contributed greatly to what we know regarding examining the importance of nutrition for athletes. However, a great deal of additional research is needed, especially as it relates to the consumption of food! It is hoped that these papers will convince basic and applied sports nutritionists alike to conduct additional research in these areas.

## References

[1] Close GL, Kasper AM, Morton JP. From paper to podium: quantifying the translational potential of performance nutrition research. Sports Med. 2018 (Suppl). 10.1007/s40279-018-1005-2. 10.1007/s40279-018-1005-2 PMC6445818 30671902

[2] Lis DM. Exit gluten-free and enter FODMAPs: a novel dietary strategy to reduce gastrointestinal symptoms in athletes. Sports Med. 2018 (Suppl). 10.1007/s40279-018-01034-0 PMC6445805 30671907

[3] Kennedy DO. Phytochemicals for improving aspects of cognitive function and psychological state potentially relevant to sports performance. Sports Med. 2018 (Suppl). 10.1007/s40279-018-1007-0. 10.1007/s40279-018-1007-0 PMC6445817 30671903

[4] Bowtel J, Kelly V. Fruit-derived polyphenol supplementation for athlete recovery and performance. Sports Med. 2018 (Suppl). 10.1007/s40279-018-0998-x. 10.1007/s40279-018-0998-x PMC6445811 30671906

[5] Burd NA, Beals JW, Martinez IG, Salvador AF, Skinner SK. Food-first approach to enhance the regulation of post-exercise skeletal muscle protein synthesis and remodelling. Sports Med. 2018 (Suppl). 10.1007/s40279-018-1009-y. 10.1007/s40279-018-1009-y PMC6445816 30671904

[6] Trangmar SJ, González-Alonso J. Heat, hydration and the human heart, muscles and brain. Sports Med. 2018 (Suppl). 10.1007/s40279-018-1033-y PMC6445826 30671905

